# Biocontrol activity and action mechanism of *Paenibacillus polymyxa* strain Nl4 against pear Valsa canker caused by *Valsa pyri*

**DOI:** 10.3389/fmicb.2022.950742

**Published:** 2022-07-22

**Authors:** Hongbo Yuan, Mengjia Yuan, Bingke Shi, Zhuoni Wang, Tianxiang Huang, Genhong Qin, Hui Hou, Li Wang, Hongtao Tu

**Affiliations:** Zhengzhou Fruit Research Institute, Chinese Academy of Agricultural Sciences, Zhengzhou, China

**Keywords:** pear Valsa canker, *Valsa pyri*, *Paenibacillus polymyxa*, biological control, antagonistic mechanism

## Abstract

Pear Valsa canker caused by *Valsa pyri* is among the most destructive diseases of pear, which causes significant economic loss. The present study was developed to explore the biocontrol efficiency and underlying antagonistic mechanism of *Paenibacillus polymyxa* strain Nl4 against *V. pyri*. *P. polymyxa* strain Nl4, one of the 120 different endophytic bacterial strains from pear branches, exhibited strong inhibitory effects against the mycelial growth of *V. pyri* and caused hyphal malformation. Culture filtrate derived from strain Nl4 was able to effectively suppress mycelial growth of *V. pyri*, and was found to exhibit strong protease, cellulase and β-1, 3-glucanase activity. Through re-isolation assay, strain Nl4 was confirmed to be capable of colonizing and surviving in pear branch. Treatment with strain NI4 effectively protected against pear Valsa canker symptoms on detached pear twigs inoculated with *V. pyri*. Moreover, strain Nl4 promoted enhanced plant growth probably through the solubilization of phosphorus. Comparative transcriptomic analyses revealed that strain NI4 was able to suppress *V. pyri* growth in large part through the regulation of the expression of membrane- and energy metabolism-related genes in this pathogen. Further transcriptomic analyses of pear trees indicated that strain NI4 inoculation was associated with changes in the expression of genes associated with secondary metabolite biosynthesis, signal transduction, and cutin, suberine, and wax biosynthesis. Together, these data highlighted *P. polymyxa* strain Nl4 as a promising biocontrol agent against pear Valsa canker and investigated the possible mechanisms of strain Nl4 on control of this devastating disease.

## Introduction

Pear Valsa canker is one of the most damaging diseases affecting pears, resulting in severe yield losses and associated economic harm to growers. This disease, which is caused by the fungus *Valsa pyri*, can cause the bark of pear trees to turn reddish-brown, soft, and rotten following infection (Wang et al., 2014; Yin et al., 2015). While fungicides are the most commonly used tools to control pear Valsa canker, food safety concerns have increasingly led to the need to limit the application of these pesticides. There is thus a clear need for the development of alternative or complementary approaches to controlling this disease, with endophyte-based biocontrol representing a particularly attractive disease control strategy.

Several different endophyte have been reported to be effective agents in the bicontrol of pear Valsa canker, such as *Bacillus subtilis*, *Bacillus velezensis*, *Bacillus amyloliquefaciens*, *Lysobacter enzymogenes*, and *Penicillium citrinum* (Cheng et al., 2017; Song et al., 2020; Liu R. et al., 2021; Yu et al., 2021; Yuan et al., 2021). *Bacillus velezensis* strains D4 and P2-1, for example, can readily inhibit *V. pyri* growth (Liu R. et al., 2021; Yuan et al., 2022), while *B. subtilis* strain 168-produced dipicolinic acid (DPA) can exert antifungal activity through the suppression of chitin synthesis (Song et al., 2020). Despite these promising results, the availability of biocontrol agents in preventing pear Valsa canker remains limited, hampering the effective control of this disease in agronomic practice.

*Paenibacillus polymyxa* is a *Bacillus* species that has previously been reported to be an effective biocontrol agent capable of inhibiting several plant diseases (Daud et al., 2019). For example, *P. polymyxa* strain HX-140 was found to readily inhibit the growth of *F. oxysporum* f. sp. *cucumerinum*, which causes cucumber Fusarium wilt (Zhai et al., 2021). Moreover, *P. polymyxa* strain JY1-5 effectively controlled tomato gray mold caused by *Botrytis cinere* (Zhang et al., 2021), while *P. polymyxa* strain APEC128 readily antagonized the development of apple anthracnose caused by *Colletotrichum gloeosporioides* and *Colletotrichum acutatum* (Kim et al., 2016). To date, however, there have not been any studies describing the use of *P. polymyxa* strains for the biocontrol of pear Valsa canker.

Prior studies have demonstrated that the induction of antifungal defense mechanisms is one of the primary mechanisms whereby *P. polymyxa* can exert its antifungal activity. For example, Liu H. et al. (2021) found that the treatment of peppers with *P. polymyxa* strain SC2 resulted in the induction of systemic responses tied to the upregulation of specific transcription factors (Liu H. et al., 2021). In cucumber roots, *P. polymyxa* strain NSY50 can similarly induce the expression of *PR1* and *PR5*, thereby conferring enhanced antifungal resistance (Du et al., 2017), while *P. polymyxa* strain AC-1 can regulate salicylic acid to coordinate the induction of plant resistance mechanisms (Hong et al., 2016). These previous studies thus highlight a range of mechanisms underlying the *P. polymyxa*-mediated biocontrol of plant diseases, providing a foundation for the use of these bacteria in agricultural contexts.

In the present study, 120 different bacterial isolates from pear branches were screened for the ability to inhibit *V. pyri* growth. Among these strains, *P. polymyxa* strain Nl4 was found to exhibit robust antifungal activity against *V. pyri* and several other pathogenic fungi. Strain Nl4 had protease, cellulose and β-1, 3-glucanase activity. In addition, *P. polymyxa* strain Nl4 was able to drive enhanced plant growth probably through phosphorus solubilization. Further analyses of the ability of this endophytic bacterial strain to colonize pear twigs were additionally conducted, while transcriptomic analyses were used to explore the potential mechanisms underlying the strain NI4-mediated biocontrol of pear Valsa canker caused *V. pyri*.

## Materials and methods

### Pathogenic fungal isolates

The *V. pyri*, *Valsa mali*, *C. gloeosporioides* and *Botryosphaeria dothidea* strains used for the present study (Yuan et al., 2022) were cultured in potato dextrose agar (PDA; potato extracts 200 g L^−1^, glucose 20 g L^−1^, and agar 15 g L^−1^) and grow at 25°C.

### Isolation and screening of potentially antagonistic bacteria

Healthy one-year-old branches were harvested in June 2021 from an “Enli” pear tree in Zhengzhou (Henan province, China) to isolate endophytic bacteria with the procedure as the same as reported previously (Yuan et al., 2022). The ability of all isolated endophytes to inhibit the growth of *V. pyri* was assessed using a dual culture screening approach as detailed previously by Yuan et al. (2022). Briefly, after overnight culture in LB broth (peptone 10 g L^−1^, yeast extract 5 g L^−1^, and sodium chloride 10 g L^−1^), 3 μl of bacteria were inoculated on PDA medium on each side of a Petri dish (2 cm from the center), with a mycelial plug (diameter: 5 mm) being placed in the center of this plate. Plates to which no bacteria were added served as controls. Plates were incubated at 25°C, and pathogen colony diameter was measured at 6 days post-inoculation (dpi). This screening assay was repeated in triplicate, with three replicates per assay. Following preliminary screening, the antifungal activity of identified antagonistic strains against other fungal pathogens (*V. mali*, *C. gloeosporioides*, and *B. dothidea*) was additionally assessed.

Hyphal morphological characterization was performed with an ultra-depth three-dimensional microscope (KEYENCE, Japan) after dual culture for 2 days. This assay was repeated three times, with at least 10 hyphae being analyzed for each replicate assay.

### Identification of antagonistic strain

Morphological and molecular approaches were employed to identify selected antagonistic endophytic bacterial strains. Morphological identification was performed as reported previously (Holt et al., 1994), while molecular identification was performed *via* the 16S rDNA sequencing of this strain using appropriate primers (Fan et al., 2016; Supplementary Table 1). After amplification, PCR products were sequenced by Bgi Genomics Co., Ltd., Beijing, China, with a BLAST comparison being used to compare these sequences in the NCBI nucleotide collection database. Similar sequences from other isolates were used to conduct multiple sequence alignment using MEGA 7.0 software, after which a phylogenetic tree was constructed with the neighbor-joining approach with 1,000 bootstrap replicates.

### Antifungal activity of culture filtrate of antagonistic strain against *Valsa pyri* mycelial growth

Culture filtrate was harvested from antagonistic bacteria following culture for 2 days at 28°C, 200 rpm. Filtrate was sterilized by passing them through a 0.22 μm filter and was combined with PDA medium at final culture filtrate concentrations of 2%, 4%, 8%, or 16%. The diameters of *V. pyri* colonies grown on PDA medium supplemented with these various culture filtrate concentrations were assessed at 6 dpi, with filtrate-free PDA serving as a control. This analysis was repeated in triplicate, with three replicates per assay.

### Secreted enzyme activity analyses

The secreted protease, cellulase, and β-1, 3-glucanase activity of strain Nl4 was, respectively, assessed using skim milk medium, CMC medium, and aniline blue medium after incubating these plates for 3 days at 28°C (Zhai et al., 2021). This analysis was repeated in triplicate with two replicates per analysis.

### Antifungal activity of *Paenibacillus polymyxa* strain Nl4 against *Valsa pyri in vivo*

The ability of *P. polymyxa* strain NI4 to control *V. pyri* infection of pear twigs (Zhongli no. 1) was assessed as reported previously with some modification (Yuan et al., 2021). Briefly, 1-year-old pear twigs were collected, rinsed with sterilized water, disinfected using 75% ethanol, and cut into 10 cm lengths. A sterile borer was used to generate a punch (diameter: 5 mm) in the center of each of these twigs, after which each twig was sprayed evenly with 1 ml of a strain Nl4 cell suspension (1 × 10^8^ CFU ml^−1^). After dry, twigs were then inoculated with *V. pyri* mycelial plugs (diameter: 5 mm). Sterile water and carbendazim (CBZ, 0.8 g L^−1^; Tianjin Hanbang Plant Protective Agent Co., Ltd., Tianjing, China) treatments, respectively, served as negative and positive controls. Following inoculation, twigs were incubated at 25°C. At 7 dpi, vernier calipers were used to measure lesion length. This analysis was repeated in triplicate, with 10 inoculation sites per replicate experiment.

### *Paenibacillus polymyxa* strain Nl4 colonization of pear twig wounds

A sterile borer was used to punch pear twigs as above, with 20 μl of strain NI4 suspension (1 × 10^8^ CFU ml^−1^) being applied to each wound. These twigs were then incubated at 25°C, with ~0.05 g of wounded tissue being harvested and ground to isolate bacterial colonies at 0 (3 h post-inoculation),1, 2, 3, 4, 5, 6, 7, 8, 9, and 10 dpi. For each sample, 0.1 ml of each prepared dilution was applied to NA medium (peptone 10 g L^−1^, beef extract 3 g L^−1^, sodium chloride 5 g L^−1^, and agar 15 g L^−1^) plates, with bacterial colonies being counted at 2 dpi following culture at 28°C. This analysis was repeated two times, with three replicates per assay.

### Effect of *Paenibacillus polymyxa* strain Nl4 on plant growth

Germinated tomato seedlings were sown in seedling pots in a growth chamber at 25°C under 60% ± 5% relative humidity with a 16 h light/8 h dark photoperiod. On day 10 after transplantation, these seedlings were irrigated with a 5 ml suspension of strain NI4 (1 × 10^8^ CFU ml^−1^) at a range of concentrations (1×, 10×, 50×). An equivalent volume of LB broth medium served as a negative control. Tomato plant growth was assessed at 10 dpi based on plant height, fresh weight, and dry weight. This analysis was repeated in triplicate, with six plants per replicate.

### Potential plant growth promoting traits of *Paenibacillus polymyxa* strain Nl4

Indole acetic acid (IAA) production was detected using Salkowski colorimetric method (Glickmann and Dessaux, 1995). Strain Nl4 was inoculated in 20 ml LB broth supplemented with 0.5 mg ml^−1^ l-Tryptophane at 28°C, 200 rpm. The supernatant of strain Nl4 was mixed with Salkowski reagent in a ratio of 1:2 for IAA assay. Phosphate solubilization assay was performed by placing stain Nl4 on Bacterial Organo-phosphorus Medium and Norganic Phosphorus Medium purchased from Hope Bio-Tcehnology Co., Ltd. (Qingdao, China) for growth 3 days.

### Transcriptome analysis

To examine changes in global *V. pyri* gene expression in response to antagonistic bacteria, wild-type *V. pyri* strain lfl-XJ was harvested from PDA medium to extract RNA following dual culture for 2 days with strain NI4, as above. *V. pyri* grown on PDA medium in the absence of strain NI4 served as a control for these analyses. To assess strain NI4 treatment-associated changes in global gene expression in pear twigs, healthy twigs were sprayed with a suspension of strain NI4 (1 × 10^8^ CFU ml^−1^), and bark samples were collected from these twigs on day two post-spraying. LB-treated pear twigs served as a control for these analyses. Trizol was used to extract total RNA from these samples based on provided directions (TransGen Biotech, Beijing, China), after which transcriptomic sequencing and downstream analyses were performed by Nanjing Personalbia Gene Technology Co., Ltd. (Nanjing, Jiangsu, China). A NEB Next Ultra Directional RNA Library Prep Kit for Illumina (NEB, CA, United States) was used to prepare sequencing libraries, while an Illumina Novaseq 6000 instrument was used for sequencing. Differentially expressed genes (DEGs) were those exhibiting a fold change |log_2_ (fold change)| > 1 and an adjusted *p* < 0.05. Gene ontology (GO) annotations were assessed with the topGO software (Alexa and Rahnenfuhrer, 2010), while KEGG pathway enrichment analyses were conducted with the clusterProfiler package (Yu et al., 2012).

### Quantitative real-time PCR assay

A quantitative real-time PCR (qRT-PCR) approach was used to validate RNA-seq results by analyzing the expression of several randomly selected genes. Samples were collected as the same as above, after which first-strand cDNA was prepared with the MonScript™ RTIII All-in-One Mix with dsDNase Kit (Monad, Suzhou, China). All qPCR analyses were performed with the Light CyclerR 96 PCR Detection System and a ChemoHS qPCR Kit (Monad, Suzhou, China) based on provided directions using the primers listed in Supplementary Table 1. The *Ppactin* and *Vpactin* genes served as normalization controls for analyses of pear and *V. pyri* gene expression, respectively, with the 2^−ΔΔCt^ method (Livak and Schmittgen, 2001) being used to quantify relative gene expression. Analyses were repeated two times with three replicates per analysis.

## Results

### Antagonistic strain screening and isolation

**Figure 1:**
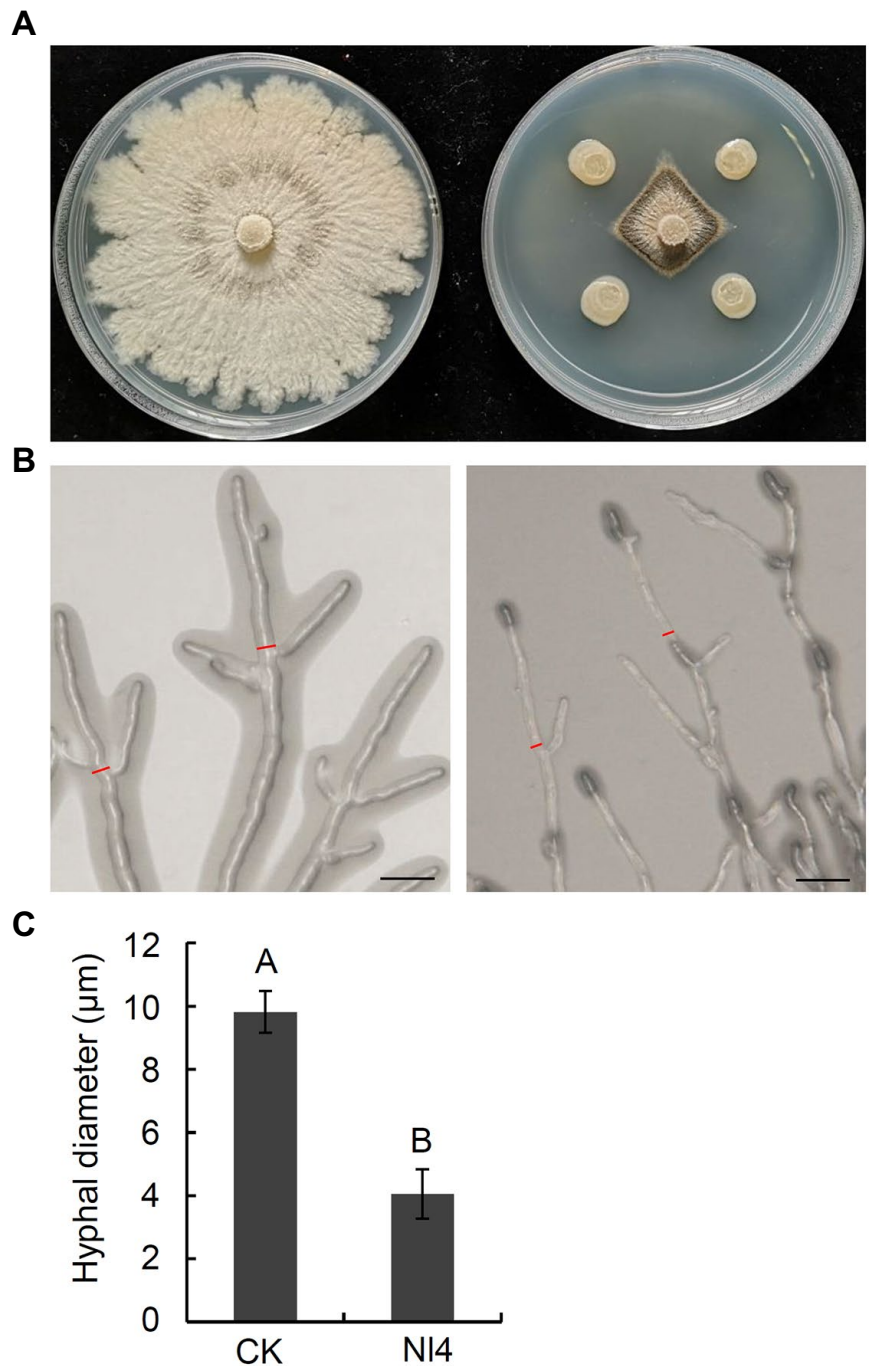
Antifungal activity of strain Nl4 against *Valsa pyri*. **(A)** Effect of strain Nl4 on mycelial growth of *V. pyri*. The picture was taken at 6 dpi. Left, *V. pyri* CK; Right, *V. pyri* antagonized with strain Nl4. **(B)** Effect of strain Nl4 on hyphal morphology of *V. pyri*. Hyphal morphology of *V. pyri* was observed at 2 days after antagonism with strain Nl4. Bar = 20 mm. **(C)** Hyhal diameter of *V. pyri*. Values are means ± SD from three replicates. The measure position of hyphae diameter was indicated in **(B)** with red line. Different uppercase letters indicate significant differences between CK and treatment according to Student’s *t*-test (*p* < 0.01).

In total, 120 bacteria were isolated from pear branches. The majority of these bacterial strains did not exhibit clear inhibition zones in dual culture tests with *V. pyri* (Supplementary Figure 1), with just five strains exhibiting significant antagonistic activity. Of these strains, stain NI4 exhibited the most robust antagonist activity, inhibiting ~75.9% of *V. pyri* mycelial growth (Figure 1A), with a 7.8 mm inhibition zone. Further analyses of the impact of strain NI4 on *V. pyri* hyphae revealed that these hyphae were abnormally stretched and deformed with a black shadow-like appearance in the context of strain NI4-mediated inhibition (Figure 1B). Statistical analyses suggested that the average *V. pyri* hyphal diameter that grown in the presence of strain NI4 was just 4.1 μm, which was significantly reduced relative to that observed for control *V. pyri* (Figure 1C).

The ability of strain NI4 to inhibit the growth of three other major fruit pathogens was also measured, revealing that this bacterium were able to significantly inhibit *V. mali*, *B. dothidea*, and *C. gloeosporioides* growth by 74.6%, 74.9%, and 69.8%, respectively. Together, these data thus suggested that strain NI4 exhibited broad-spectrum antifungal activity *in vitro*.

### Strain Nl4 culture filtrate suppressed *Valsa pyri* growth

**Figure 2:**
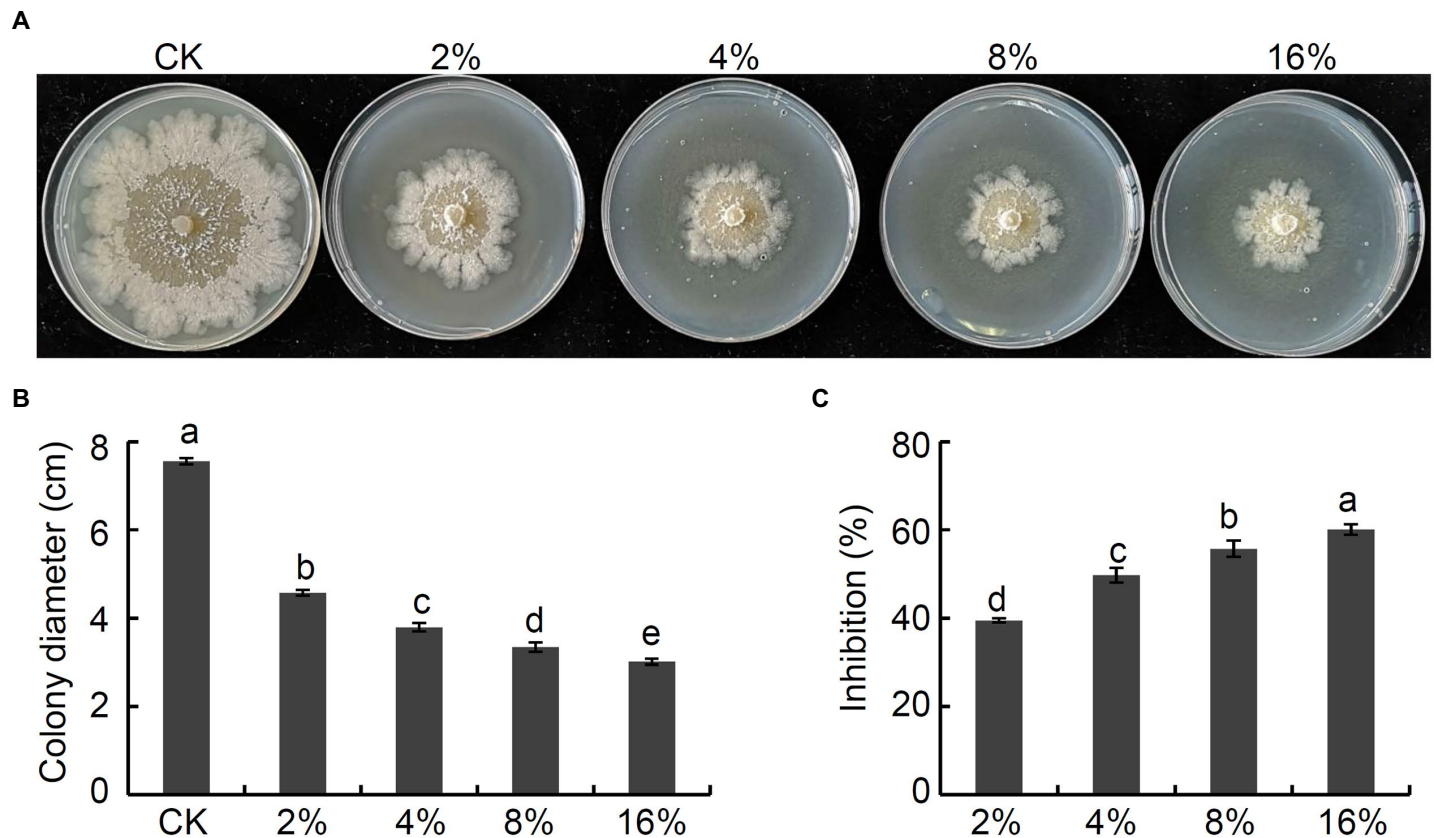
Antifungal activity of culture filtrate of strain Nl4 against *V. pyri*. **(A)** Effect of culture filtrate of strain Nl4 on mycelial growth of *V. pyri*. The picture was taken at 6 dpi. **(B)** Statistical analysis of colony diameter. **(C)** Inhibition of strain Nl4 culture filtrate on mycelial growth of *V. pyri*. Each data represents the mean ± SD of three replicates. Letters above the bars indicate statistical significance and different lowercase letters indicate significant different means (*p* < 0.05) based on Student’s t-test.

To establish whether culture filtrate prepared from strain NI4 would similarly possess antagonistic activity against *V. pyri*, culture filtrate of strain Nl4 were added to PDA medium at final concentrations of 2%, 4%, 8%, or 16%. Subsequent results demonstrated that these strain NI4 filtrate significantly inhibited *V. pyri* mycelial growth in a dose-dependent fashion (Figures 2A,B). Specifically, the inhibition of *V. pyri* mediated by 2%, 4%, 8%, and 16% culture filtrate preparations were 39.5%, 49.7%, 55.7%, and 60.1%, respectively (Figure 2C). As such, culture filtrate prepared from strain NI4 exhibited robust antifungal activity against *V. pyri*.

### Analysis of the secreted enzyme activity of strain Nl4

**Figure 3:**
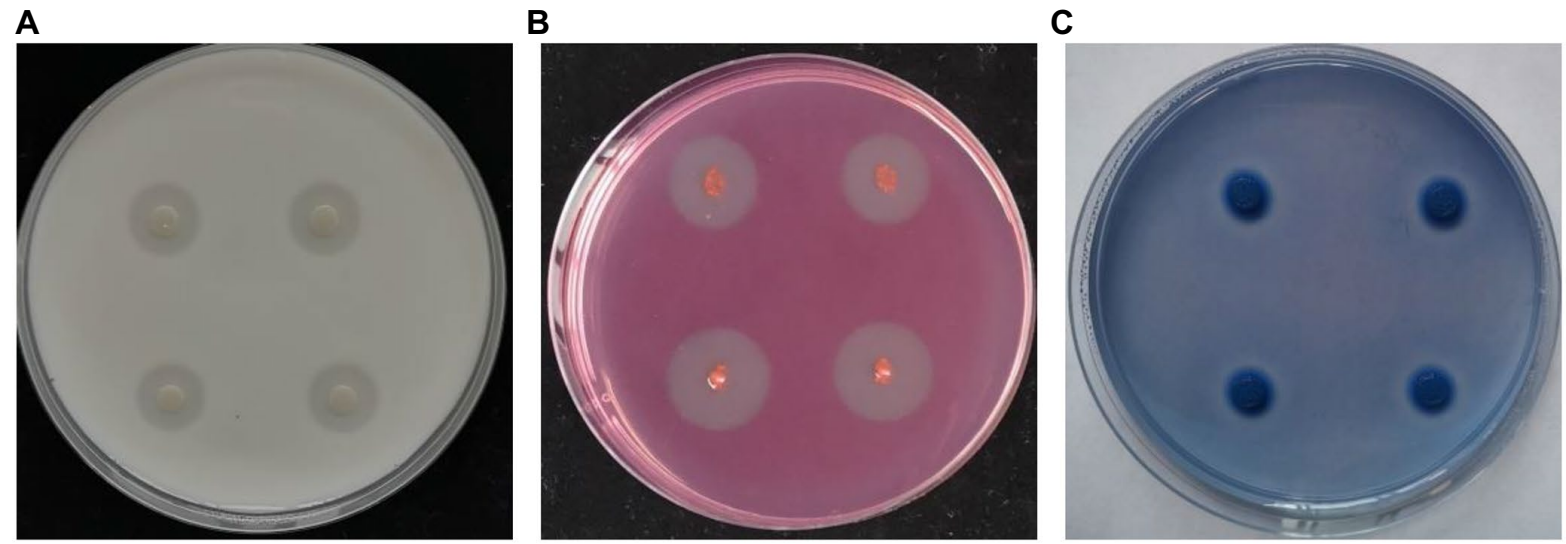
Secreted enzyme activity of strain Nl4. Protease **(A)**, cellulose **(B)**, and β-1, 3-glucanase **(C)** activities of strain Nl4 were analyzed on skim milk medium, CMC medium, and aniline blue medium, respectively. The pictures were taken at 3 dpi at 28°C.

Next, secreted enzyme activity analyses were conducted for strain NI4, revealing that it was capable of forming a clear transparent circle on skim milk medium, CMC medium, and aniline blue medium (Figure 3). These results thus indicated that enzymes or metabolites produced by strain NI4 exhibited protease, cellulase, and β-1, 3-glucanase activity, potentially accounting for the ability of strain Nl4 to degrade *V. pyri* hyphae.

### Identification of strain Nl4

Biochemical and physiological analyses of strain NI4 were next performed (Supplementary Table 2). This bacterium was identified as a gram-positive strain that yielded positive Voges-Proskauer (VP), nitrate reductase, starch hydrolysis, and gelatin liquefaction test results and a negative citrate test (Supplementary Table 2). Strain Nl4 was capable of growing on media containing sucrose, xylose, or mannitol as a carbon source, but could not grow on medium containing more than 5% sodium chloride.

**Figure 4:**
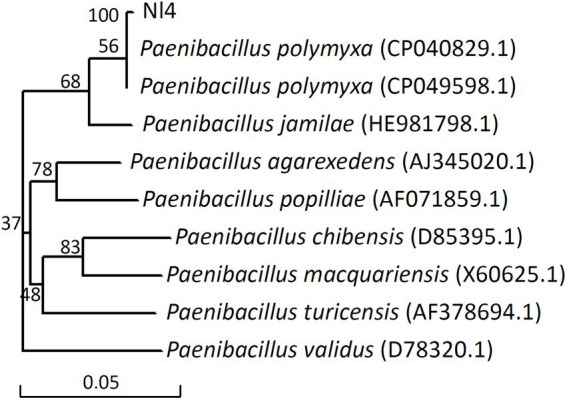
Phylogenetic analysis of strain Nl4 and its relatives based on 16S rDNA.

A phylogenetic tree constructed based on the partial 16S rDNA sequence of strain NI4 (accession number: ON763838), together with closely related sequences, suggested this strain to be most closely related to *P. polymyxa* (Figure 4). As such, strain Nl4 was identified as *P. polymyxa*.

### Colonization of *Paenibacillus polymyxa* strain Nl4 in pear twigs wounds

**Figure 5:**
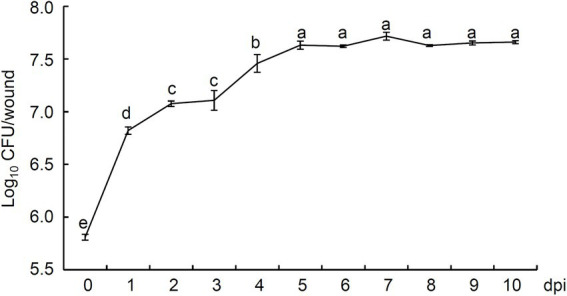
Population dynamics of *Paenibacillus polymyxa* strain Nl4 in wounds of pear twig. Population densities were expressed as Log_10_ CFU/wound. Each data represents the mean ± SD of three biological replicates. Different lowercase letters indicate significant differences according to Student’s *t*-test (*p* < 0.05).

At 0 dpi, 6.47 × 10^5^ CFU/wound strain NI4 colonies were observed in wound sites on pear twigs, rapidly expanding over 10-fold to 6.63 × 10^6^ CFU at 1 dpi (Figure 5). The peak number of NI4 colonies was observed in pear branches at 5 dpi (4.28 × 10^7^ CFU; 66.19-fold higher than 0 dpi), after which the number of colonies remained largely consistent with slight fluctuations (Figure 5). At 10 dpi, a high number of strain Nl4 colonies was still present in pear twig wound sites (Figure 5). These results thus demonstrated the ability of strain NI4 to readily colonize wounded pear twigs.

### Antifungal activity of *Paenibacillus polymyxa* strain Nl4 against *Valsa pyri in vivo*

**Figure 6:**
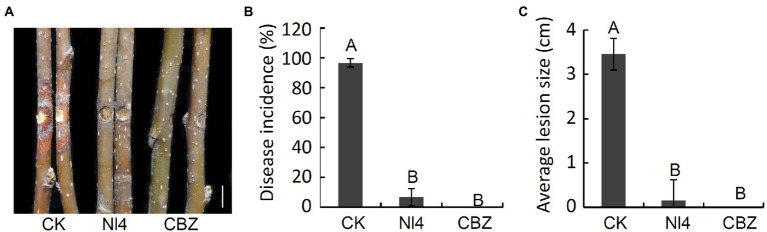
Biocontrol activity of *P. polymyxa* strain Nl4 in controlling pear Valsa canker caused by *V. pyri*. **(A)** Suppression of strain Nl4 cell suspension on pear Valsa canker. Sterile distilled water or carbendazim (CBZ) treatment was used as the negative and positive control, respectively. Bar = 1 cm. **(B)** Statistical analysis of the disease incidence. **(C)** Statistical analysis of disease lesion size. Each data represents the mean ± SD of three replicates. Disease lesion size and disease incidence were measured at 7 dpi. Letters above the bars indicate statistical significance and different uppercase letters indicate significant different means (*p* < 0.01) based on Student’s t-test.

To assess the ability of *P. polymyxa* strain NI4 to inhibit the development of pear Valsa canker caused by *V. pyri*, a suspension of NI4 was applied to pear twigs that were then inoculated with *V. pyri*. On day 7 following *V. pyri* inoculation, control pear twigs exhibited brown lesions at the inoculated site, while twigs that had been treated with a suspension of strain NI4 cells exhibited less symptoms of disease (Figure 6A). Overall, strain NI4 treatment was associated with a reduction in disease incidence to just 6.7% as compared to the ~96.7% disease incidence observed in control twigs (Figure 6B). Pear twigs treated with CBZ as a positive control remained free of disease (Figures 6A,B). Average lesion size values were also significantly reduced in treated pear twigs relative to those observed on control twigs (Figure 6C). These data thus supported the ability of *P. polymyxa* strain Nl4 to suppress the development and severity of pear Valsa canker caused by *V. pyri*.

### PGP traits of *Paenibacillus polymyxa* strain Nl4

To explore the plant growth promoting (PGP) characteristics of *P. polymyxa* strain NI4, different proportions of strain NI4 cell suspensions were applied to tomato seedling roots. As shown in Table 1, strain NI4 treatment was associated with significant increases in plant height, with plants treated with 1x, 10x, and 50x strain NI4 cell suspensions exhibiting respective heights of 10.18, 10.94, and 10.48 cm, respectively, as compared to a control plant height of just 7.54 cm (Table 1). An increase in total plant biomass was similarly observed following strain NI4 treatment (Table 1). Further results showed that strain Nl4 could not produce IAA but had the ability to dissolve organo-phosphorus (Supplementary Figure 2).

**Table 1:** Effect of *P. polymyxa* strain Nl4 on tomato growth promotion.

Treatment	Plant height (cm)	Fresh weight (g)	Dry weight (g)
CK	7.54 ± 0.63b	0.54 ± 0.21b	0.038 ± 0.016b
1 × Nl4	10.18 ± 0.77a	0.87 ± 0.15a	0.056 ± 0.004a
10 × Nl4	10.94 ± 0.36a	0.96 ± 0.13a	0.0619 ± 0.010a
50 × Nl4	10.48 ± 0.80a	0.94 ± 0.20a	0.0562 ± 0.008a

Data represents the mean ± SD of six biological replicates. Different lowercase letters indicate significant differences according to Student’s *t*-test (*p* < 0.05).

### Transcriptome analysis of *Valsa pyri* treated with *Paenibacillus polymyxa* strain Nl4

**Figure 7:**
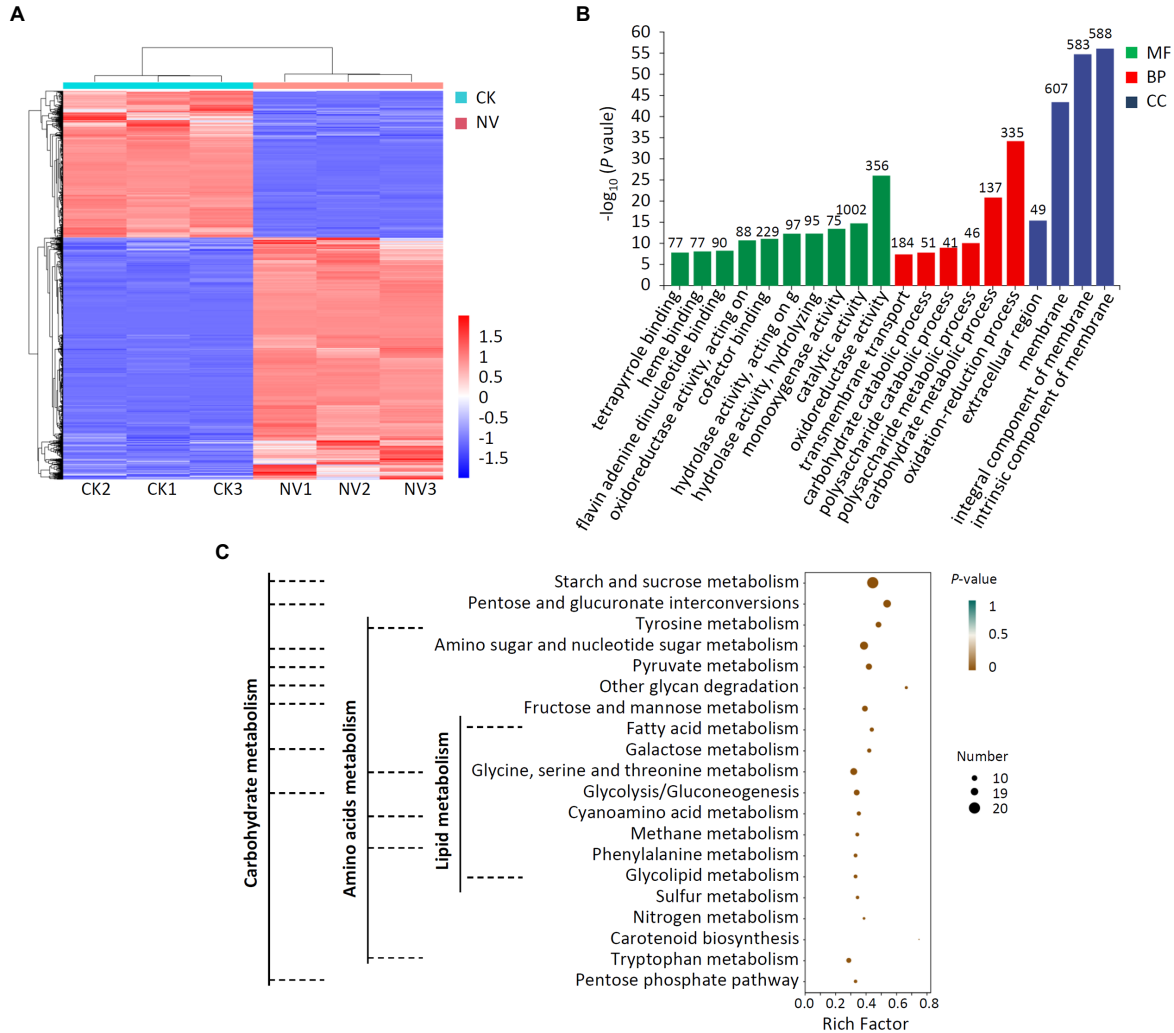
Transcriptome analysis of DEGs in *V. pyri* treated with *P. polymyxa* strain Nl4. **(A)** Heat map analysis. CK, untreated *V. pyri*; NV, strain Nl4 treated *V. pyri*. Red indicates highly expressed genes, and blue indicates low expressed genes. **(B)** GO annotation analyses of DEGs. The number above the column represents the number of DEGs. **(C)** KEGG pathway enrichment analyses of the DEGs. Rich factor = DEGs number/total gene number identified from transcriptome of a certain process.

Next, transcriptome analyses of *V. pyri* that were or were not exposed to *P. polymyxa* strain NI4 were conducted, with an *R*^2^ of 0.816–1.000 among treatments (Supplementary Figure 3), reaffirming the reproducibility of the data derived from this analysis. In total, comparisons of control and *P. polymyxa* strain NI4-treated *V. pyri* revealed 2,585 DEGs of which 1,610 and 975 were, respectively, upregulated and downregulated (Figure 7A; Supplementary Figure 4). GO analyses indicated that these DEGs were primarily associated with molecular functions including oxidoreductase activity and catalytic activity, biological processes including oxidation–reduction and carbohydrate metabolic processes, and cellular component terms including intrinsic component of membrane, integral component of membrane and membrane among cellular component (Figure 7B). Subsequent analysis indicated that 1,002 of these DEGs were associated with the catalytic activity annotation while 1,778 were associated with the membrane component annotation. KEGG analyses further revealed these DEGs to be enriched for the biosynthesis and metabolism of multiple carbohydrates, amino acids, and lipids (Figure 7C).

Further analysis indicated that the fatty acid metabolism, biosynthesis of unsaturated fatty acids, glycerophospholipid metabolism, glycosphingolipid biosynthesis and steroid biosynthesis, which associated with cell membrane, were enriched in *V. pyri* at the stress of strain Nl4. Among them, eight genes (*VP1G_09796*, *VP1G_00957*, *VP1G_08690*, *VP1G_05245*, *VP1G_07256*, *VP1G_08986*, *VP1G_04775* and *VP1G_11014*) involved in fatty acid metabolism, three genes (*VP1G_00733*, *VP1G_03079* and *VP1G_08598*) involved in unsaturated fatty acids biosynthesis, three genes (*VP1G_06941*, *VP1G_04418* and *VP1G_09142*) involved in glycerophospholipid metabolism, one gene (*VP1G_07179*) involved in glycosphingolipid biosynthesis and two genes (*VP1G_04184* and *VP1G_04418*) involved in steroid biosynthesis were downregulated. In order to alleviate oxidative damages of strain Nl4 on the cell membrance, plenty of genes involved in antioxidant response were significantly activated in *V. pyri*. For example, the genes *VP1G_01209* encoding catalase-related peroxidase and *VP1G_08485* encoding L-ascorbate peroxidase were greatly upregulated. In addition, homolog genes of glutathione S-transferase (GST, *VP1G_08389*, *VP1G_09397*, *VP1G_05587* and *VP1G_04014*) and ABC transporter proteins (*VP1G_01031* and *VP1G_05741*) were also significantly upregulated, to eliminate ROS stress.

These data suggested that *P. polymyxa* strain Nl4 was able to suppress *V. pyri* growth primarily *via* impacting the membrane and energy metabolism activity of this pathogen.

### Transcriptome analysis of pear tree treated with *Paenibacillus polymyxa* strain Nl4

**Figure 8:**
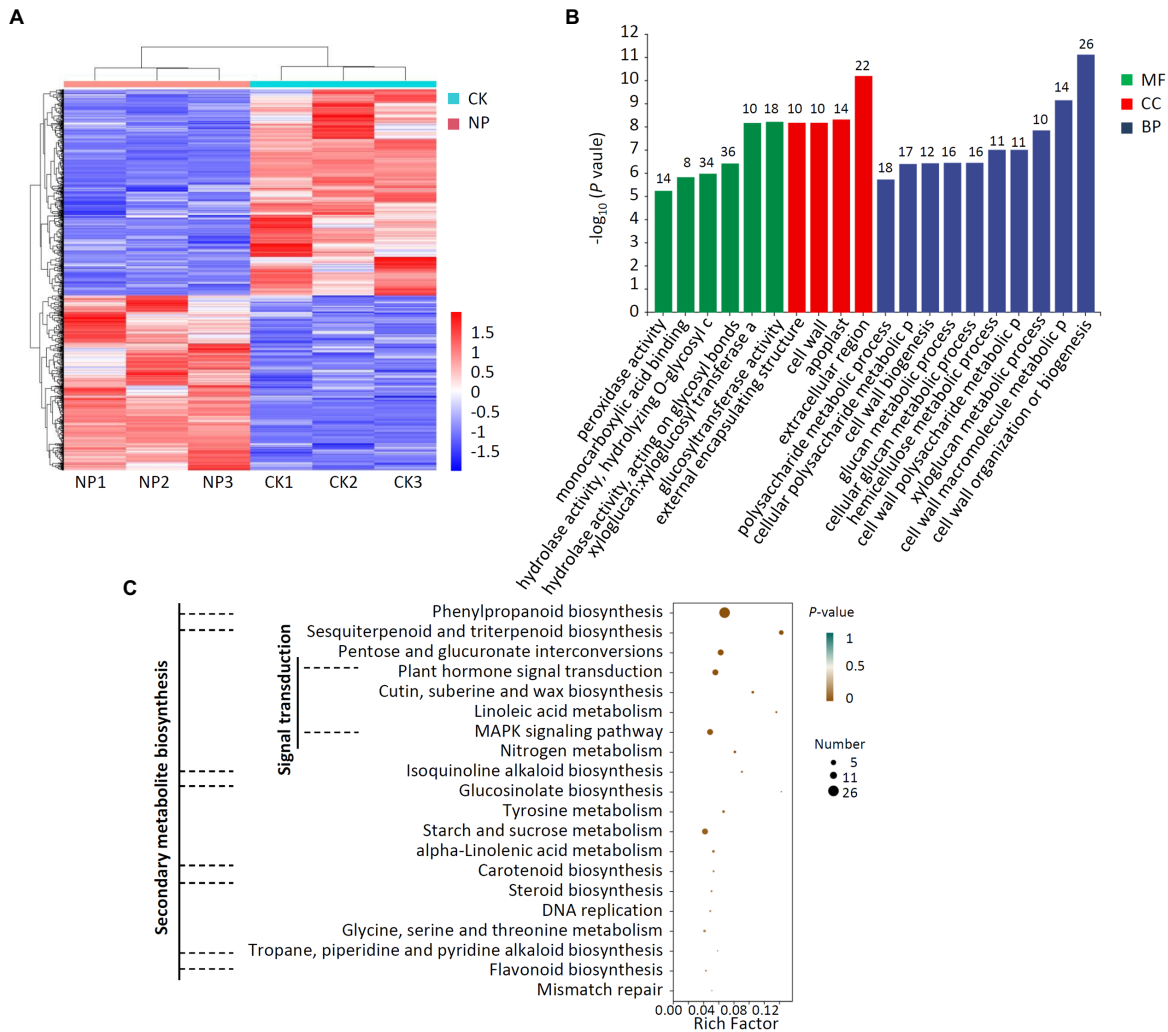
Transcriptome analysis of DEGs in pear twigs treated with *P. polymyxa* strain Nl4. **(A)** Heat map analysis. CK, untreated pear twigs; NP, strain Nl4 treated pear twigs. Red indicates highly expressed genes, and blue indicates low expressed genes. **(B)** GO annotation analyses of DEGs. The number above the column represents the number of DEGs. **(C)** KEGG pathway enrichment analyses of the DEGs. Rich factor = DEGs number/total gene number identified from transcriptome of a certain process.

A transcriptomic analysis of pear trees treated with *P. polymyxa* strain Nl4 was additionally conducted, revealing 396 and 466 DEGs that were, respectively, upregulated and downregulated following such treatment relative to control tree samples (Figure 8A; Supplementary Figure 5). GO analyses revealed these DEGs to be mainly associated with 20 different annotated subcategories, with many of these genes being enriched for GO terms pertaining to hydrolase activity, acting on glycosyl bonds, hydrolyzing O-glycosyl compounds, regulating cell wall organization or biogenesis, and impacting the extracellular region (Figure 8B). KEGG enrichment analyses further revealed these DEGs to be enriched for secondary metabolite biosynthesis pathways including phenylpropanoid biosynthesis, sesquiterpenoid and triterpenoid biosynthesis, isoquinoline alkaloid biosynthesis, glucosinolate biosynthesis, carotenoid biosynthesis, steroid biosynthesis, tropane, piperidine and pyridine alkaloid biosynthesis, and flavonoid biosynthesis (Figure 8C). Moreover, these DEGs were also enriched for cutin, suberine, and wax biosynthesis, plant hormone signal transduction, and MAPK signaling pathway activity.

After *P. polymyxa* strain Nl4 treatment, 33 genes in pear related to secondary metabolism were upregulated by 1.02–4.65 times by comparing with the control (Table 2). Some potential defense-related genes were also upregulated in pear after Nl4 treatment (Table 3). In addition, the expression of several transcription factors involved in plant disease resistance were increased, such as homologs of *WRKY 75* (*Ppy14g1309.1* and *Ppy06g1207.1*), *WRKY 40* (*Ppy09g1858.1* and *Ppy08g0450.1*). Furthermore, some genes involved in plant hormone signal transduction were upregulated. For example, gibberellin receptor *GID1C* (*Ppy12g1496.1*) was upregulated with a 1.58-fold time. ACO homolog gene in pear (*Ppy10g0793.1*) involved in the biosynthesis of ethylene was upregulated with a 1.67-fold time.

**Table 2:** List of upregulated gene related to secondary metabolism in pear twigs at the present of *Paenibacillus polymyxa* strain Nl4.

Gene	log_2_ (fold change)	Description
*Ppy14g0888.1*	1.06	4-Coumarate-CoA ligase-like
*Ppy07g2039.1*	1.07	Caffeoyl shikimate esterase
*Ppy07g1936.1*	1.39	Caffeoyl shikimate esterase
*Ppy02g1609.1*	1.26	Peroxidase 73-like
*Ppy01g1477.1*	1.96	Peroxidase P7-like
*Ppy14g0094.1*	1.49	Peroxidase 57-like
*Ppy15g0912.1*	1.50	Peroxidase 16-like
*Ppy15g2378.1*	1.42	Peroxidase 10-like
*Ppy05g2964.1*	1.74	Polyphenol oxidase
*Ppy09g1129.1*	1.14	Polyphenol oxidase IV
*Ppy05g2960.1*	2.16	Polyphenol oxidase IV
*Ppy10g1746.1*	2.28	polyphenol oxidase
*Ppy16g0851.1*	2.17	Flavanone 3-hydroxylase-like
*Ppy11g1345.1*	3.12	Secoisolariciresinol dehydrogenase-like
*Ppy10g1848.1*	2.72	Nerolidol synthase 1-like
*Ppy10g1852.1*	3.15	(3S,6E)-Nerolidol synthase 1-like
*Ppy10g0228.1*	1.02	Beta-amyrin synthase-like
*Ppy03g1922.1*	4.65	(−)-Germacrene D synthase-like
*Ppy07g0648.1*	3.05	Squalene monooxygenase-like
*Ppy07g0087.1*	2.36	Squalene monooxygenase-like
*Ppy13g2150.1*	1.51	3-Isopropylmalate dehydratase large subunit
*Ppy06g1930.1*	2.74	Eucoanthocyanidin reductase-like
*Ppy17g0031.1*	1.23	Aspartokinase 2
*Ppy01g0522.1*	2.13	(−)-Isopiperitenone reductase-like
*Ppy13g0036.1*	1.67	Serine carboxypeptidase-like
*Ppy03g0799.1*	2.87	Probable inactive 2-oxoglutarate-dependent dioxygenase
*Ppy10g0822.1*	1.35	Cytochrome P450 94A1-like
*Ppy11g1877.1*	1.43	Cytochrome P450 CYP736A12-like
*Ppy09g1129.1*	1.14	UDP-Glycosyltransferase
*Ppy11g0487.1*	1.84	UDP-Glycosyltransferase superfamily protein
*Ppy02g0042.1*	2.85	UDP-Glycosyltransferase
*Ppy08g1972.1*	2.61	7-Deoxyloganetin glucosyltransferase-like
*Ppy12g0470.1*	1.41	Probable glycosyltransferase At5g03795

**Table 3:** List of upregulated gene related to defense-related in *Paenibacillus polymyxa* strain Nl4 treated pear twigs.

Gene	log_2_ (fold change)	Description
*Ppy05g0926.1*	2.92	Pathogenesis-related protein 1-like
*Ppy05g0923.1*	1.46	Basic form of pathogenesis-related protein 1-like
*Ppy04g0597.1*	1.34	Pathogenesis-related protein 5-like
*Ppy06g0674.1*	1.88	Pathogenesis-related protein 5-like
*Ppy13g1587.1*	1.74	Pathogenesis-related protein 10
*Ppy13g1585.1*	1.37	Pathogenesis-related protein 10b
*Ppy08g1541.1*	1.36	Pathogenesis-related protein 10b
*Ppy06g1893.1*	1.14	Protein SGT1-like
*Ppy14g1921.1*	1.26	Protein SGT1-like
*Ppy07g0357.1*	1.02	TMV resistance protein N-like
*Ppy10g1810.1*	1.67	Disease resistance RPP13-like protein 4
*Ppy00g1277.1*	1.34	Salicylic acid methyltransferase
*Ppy00g0673.1*	2.00	Salicylic acid methyltransferase
*Ppy11g1304.1*	1.53	Ankyrin repeat-containing protein NPR4-like
*Ppy11g1309.1*	1.99	Ankyrin repeat-containing protein NPR4-like

Together, these results suggest that *P. polymyxa* strain Nl4 can regulate pear resistance by mainly altering the expression of resistance-related genes associated with secondary metabolite biosynthesis, signal transduction, and cutin, suberine, and wax biosynthesis.

### Validation the results of RNA-sequencing by qRT-PCR analysis

**Figure 9:**
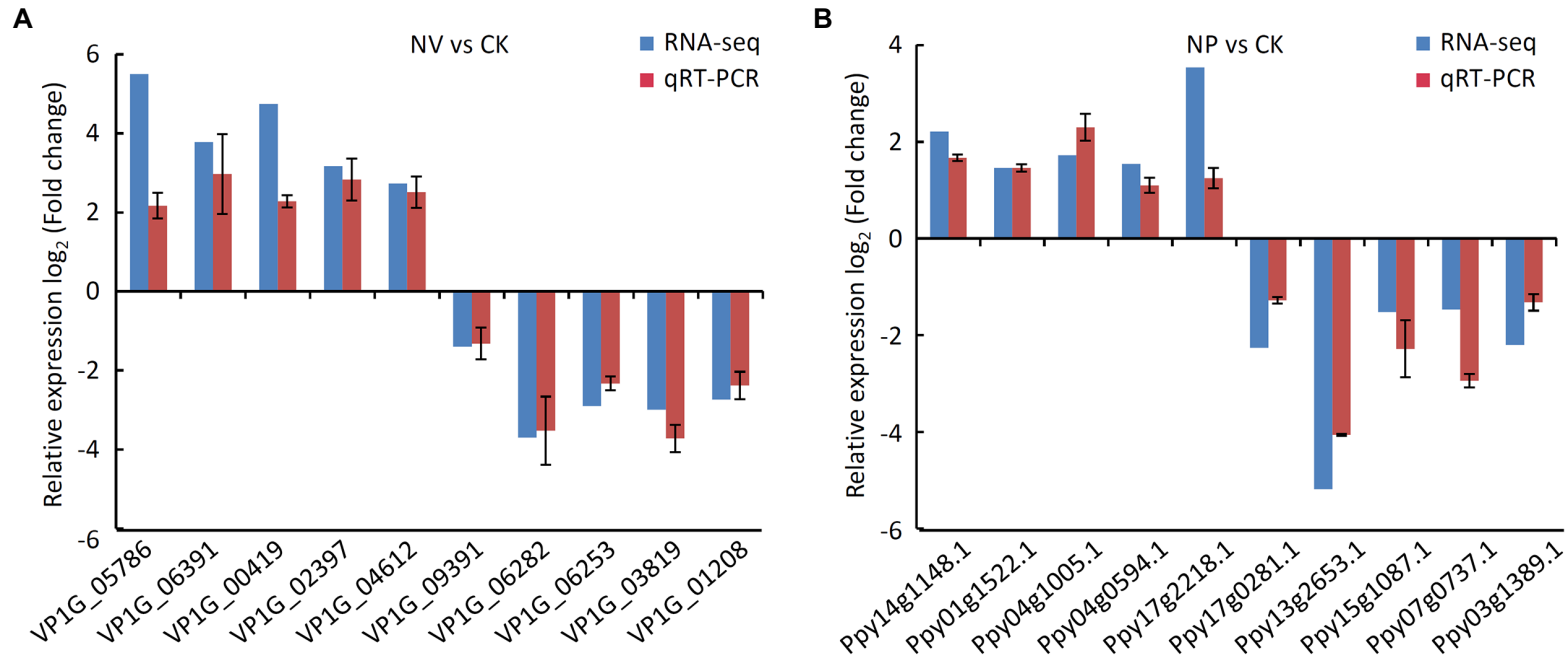
Validation the results of RNA-seq by qRT-PCR assay. **(A)** qRT-PCR assay in *V. pyri*. CK, untreated *V. pyri*; NV, strain Nl4-treated *V. pyri*. **(B)** qRT-PCR assay in pear twigs. CK, LB-treated pear twigs; NP, strain Nl4-treated pear twigs.

The result of qRT-PCR analysis showed that the expression of 10 randomly selected genes exhibited a similar expression pattern with RNA-sequencing (Figure 9), which suggested the transcriptome data in this study were reliable.

## Discussion

Pear Valsa canker is a devastating disease caused by *V. pyri* that can adversely impact pear tree yields. Endophyte-based biocontrol strategies of this disease represent an attractive alternative to chemical fungicide application, and prior studies have demonstrated the promise of such approaches (Song et al., 2020; Yuan et al., 2021). However, relatively few biocontrol strains with antagonistic activity against pear Valsa canker have been described to date. As such, in the present study, 120 bacteria were isolated from pear branches and screened for the ability to inhibit *V. pyri* growth in a dual culture test. Of the isolated strains, *P. polymyxa* strain Nl4 was most readily able to inhibit this fungal pathogen, suppressing *V. pyri* mycelial growth and inducing hyphal malformations. Notably, *P. polymyxa* strain Nl4 also exhibited broad-spectrum antifungal activity, inhibiting the growth of a range of plant pathogens in line with prior studies (Zhang et al., 2018; Slama et al., 2019). This endophytic bacterial strain was able to significantly reduce pear Valsa canker disease incidence and lesion size, exhibiting efficacy similar to that for positive control CBZ treatment. Together, these findings suggested that *P. polymyxa* strain Nl4 represents a promising biocontrol resource with the potential to combat *V. pyri*-induced pear Valsa canker.

The ability of biocontrol strains to colonize and survive in host plant tissues is critical to their agronomic utility. *P. polymyxa* strain Nl4 was initially isolated from pear branches, and it was also successfully re-isolated after being used to inoculate pear twigs. Moreover, strain NI4 rapidly proliferated after application to pear twigs, reaching peak density at 5 dpi with bacterial counts were 66.19-fold higher than at 0 dpi, after which this population remained stable throughout the remainder of the study period. These data thus indicate that *P. polymyxa* strain NI4 can readily colonize and survive on pear twigs, emphasizing the need for further studies exploring the impact of different environmental variables on this activity.

*P. polymyxa* is capable of promoting plant growth, primarily *via* nitrogen fixation (Puri et al., 2016), phosphate solubilization (Mohd Din et al., 2020), IAA (Mei et al., 2014), iron acquisition (Zhou et al., 2016), or improvements in chlorophyll content (Liu H. et al., 2021). PGP train analyses in the present study suggested that *P. polymyxa* strain Nl4 was able to solubilize phosphorus, suggesting that this may be the mechanism underlying the ability of this biocontrol agent to accelerate plant growth.

Cell wall disruption can adversely impact fungal cell growth and morphological characteristics, potentially promoting cell death (Bowman and Free, 2006). Given their importance, cell walls commonly serve as targets for antifungal treatment. Here, strain NI4 culture filtrate was found to mediate antifungal activity against *V. pyri*, suggesting that the culture filtrate contained antimicrobial compounds. Secretions produced by strain NI4 also exhibited robust protease, cellulase, and β-1, 3-glucanase activity, with all three of these enzymes being capable of breaking down fungal cell walls (Park et al., 2012; Zhai et al., 2021). Cell membranes are important for the cell to cope with different environment stress, such as chemical substances. Previous study had proven that components of fungal cell membrane, such as ergosterol was one of the main targets of antifungal agent (Jordá and Puig, 2020). In this study, transcriptomic analyses revealed that 1,778 total DEGs that were expressed in *V. pyri* in response to strain NI4 exposure were annotated as being associated with the cell membrane, suggesting that the *V. pyri* membrane may be a primary target of *P. polymyxa* strain Nl4. Strain Nl4-induced damage also caused 1,385 DEGs exhibiting oxidoreductase activity and catalytic activity in *V. pyri*. Together, these data thus suggest that the antifungal activity of *P. polymyxa* strain Nl4 against *V. pyri* may be related to its ability to inhibit cell wall and membrane synthesis, in line with similar antagonistic mechanisms that have previously been reported for other strains exhibiting antifungal activity (Yang et al., 2020; Liu R. et al., 2021).

A number of plant secondary metabolites have been shown to contribute to plant resistance (Bennett and Wallsgrove, 1994; Piasecka et al., 2015). In transcriptomic analyses of pear trees treated with *P. polymyxa* strain NI4, many of the identified DEGs were enriched in secondary metabolite biosynthesis pathways including the phenylpropanoid biosynthesis, sesquiterpenoid and triterpenoid biosynthesis, and isoquinoline alkaloid biosynthesis pathways. Phenylpropanoids are synthesized from phenylalanine, and can enable plants to resist a range of abiotic and biotic stressors (Geng et al., 2020; Yadav et al., 2020). Chen et al. (2021) found that *Pichia galeiformis* was capable of enhancing postharvest citrus resistance to the pathogen *P. digitatum via* the activation of the phenylpropanoid biosynthesis pathway. Here, 16 DEGs in pear trees were identified to associate with phenylpropanoid biosynthesis pathway following strain Nl4 treatment. Among them, 4-coumarate-CoA ligase (4CL, *Ppy14g0888.1*) is a rate-limiting enzyme involved in the phenylpropanoid metabolism to produce flavonoids, cinnamate and lignin (Hu et al., 2010). The flavonoid biosynthesis pathway is a branch of the phenylpropanoid biosynthesis pathway, with flavonoids serving as inducible phytoalexins that confer resistance to pathogens (Samanta et al., 2011; Nemesio-Gorriz et al., 2016; Ullah et al., 2017). In the present study, flavonoid biosynthesis was similarly enriched in pear twigs following strain NI4 treatment, with a 2.74-fold and 2.17-fold increase in leucoanthocyanidin reductase (LAR, *Ppy06g1930.1*) and flavanone 3-hydroxylase-like (F3H, *Ppy16g0851.1*) gene expression, respectively.

Lignin and lignan as a class of secondary metabolites belong to phenylpropane derivatives. Many studies have confirmed that lignin is an inducible physical barrier against pathogen infection by reinforcing plant cell wall (Shadle et al., 2003). Peroxidase (POD) is a key enzyme involved in lignin formation (Ali et al., 2006). In this study, we found that the expression of five PODs genes (*Ppy02g1609.1*, *Ppy01g1477.1*, *Ppy14g0094.1*, *Ppy15g0912.1* and *Ppy15g0912.1*) was significantly increased in *P. polymyxa* strain Nl4 treated pear when compared with control. Other study also found a similar result in citrus treated with antagonistic strain (Chen et al., 2021). Gene encoding caffeoyl shikimate esterase-like (CSE, *Ppy07g2039.1* and *Ppy07g1936.1*), as an enzyme function in the lignin biosynthetic pathway through hydrolyzing caffeoyl shikimate into caffeate (Vanholme et al., 2013), were also upregulated. In addition, Gene encoding secoisolariciresinol dehydrogenase-like (SDH, *Ppy11g1345.1*) involved in the biosynthesis of lignan showed higher upregulation by increasing to 3.12-fold.

CYP450s and glycosyltransferase (UGTs) are considered to be involved in the biosynthesis of triterpene saponins that participate in plant defense (Rahimi et al., 2019). CYP450s can catalyze the carboxylation, hydroxylation, dehydrodehydration, alkylation and kekylation of triterpene backbones to form the intermediate of triterpene saponins (Thimmappa et al., 2014). UGTs function at the last step in triterpene saponin biosynthesis by glycosylation (Augustin et al., 2012). In this study, two CYP450s genes (*Ppy10g0822.1* and *Ppy11g1877.1*) and five UGTs genes (*Ppy09g1129.1*, *Ppy11g0487.1*, *Ppy02g0042.1*, *Ppy08g1972.1* and *Ppy12g0470.1*) were upregulated in pear. Other plant secondary metabolites regulated by strain NI4 including sesquiterpenoids and isoquinoline alkaloids can also confer pathogen resistance (Bennett and Wallsgrove, 1994; Morrissey, 2009). Together, these data suggest that *P. polymyxa* strain Nl4 can induce pear antifungal defenses primarily through the regulation of secondary metabolite biosynthesis pathways.

## Conclusion

In conclusion, *P. polymyxa* Nl4 was herein identified as a promising biocontrol agent capable of preventing pear Valsa canker caused by *V. pyri*. Preliminary transcriptomic analyses were conducted to explore the mechanisms whereby *P. polymyxa* strain NI4 can suppress *V. pyri* growth and induce plant defense responses, providing a robust foundation for future efforts to apply this bacterium as a good biocontrol agent.

## Data availability statement

The data presented in the study are deposited in NCBI, accession number PRJNA851571 (https://www.ncbi.nlm.nih.gov/sra/PRJNA851571) and PRJNA851531 (https://www.ncbi.nlm.nih.gov/sra/PRJNA851531).

## Author contributions

HY and HT designed the research. HY, MY, BS, and ZW performed the experiments with help from TH, GQ, HH, and LW. HY analyzed the data and wrote the manuscript. HT provided the funding. All authors contributed to the article and approved the submitted version.

## Funding

This work obtained the financial support of the National Key R&D Program of China (no. 2017YFE0135600), the Agricultural Science and Technology Innovation Program (CAAS-ASTIP-2016-RIP), and the Central Public-Interest Scientific Institution Basal Research Fund (no. ZGS202110).

## Conflict of interest

The authors declare that the research was conducted in the absence of any commercial or financial relationships that could be construed as a potential conflict of interest.

## Publisher’s note

All claims expressed in this article are solely those of the authors and do not necessarily represent those of their affiliated organizations, or those of the publisher, the editors and the reviewers. Any product that may be evaluated in this article, or claim that may be made by its manufacturer, is not guaranteed or endorsed by the publisher.

## Supplementary materials

The Supplementary materials for this article can be found online at: https://www.frontiersin.org/articles/10.3389/fmicb.2022.950742/full#supplementary-material

Supplementary Figure 1 The endophyte strain Nl7 did not show antagonistic activity against *V. pyri.*

Supplementary Figure 2 PGP traits of strain Nl4. **(A)** IAA assay. Landy medium was used as negative control and IAA (10 mg L^-1^) was used as positive control. **(B)** Phosphate solubilization assay. Left, inorganic phosphorus; Right, organo-phosphorus.

Supplementary Figure 3 Correlation heatmap between *V. pyri* CK and *V. pyri* treated with *P. polymyxa* strain Nl4 in transcriptome analysis. CK, untreated *V. pyri*; NV, strain Nl4 treated *V. pyri*.

Supplementary Figure 4 Transcriptome pattern in *V. pyri* at the present of *P. polymyxa* strain Nl4. Downregulated DEGs are shown with blue dots, while the upregulated DEGs are shown in red. Those that are not significantly altered are shown in grey in the center.

Supplementary Figure 5 Transcriptome pattern in pear twigs at the present of *P. polymyxa* strain Nl4. Downregulated DEGs are shown with blue dots, while the upregulated DEGs are shown in red. Those that are not significantly altered are shown in grey in the center.

## References

[ref1] Alexa A. Rahnenfuhrer J. (2010). TopGO: enrichment analysis for gene ontology. R package, version 2.22.0.

[ref2] Ali M. B. Khatun S. Hahn E. J. Paek K. Y. (2006). Enhancement of phenylpropanoid enzymes and lignin in *Phalaenopsis orchid* and their influence on plant acclimatisation at different levels of photosynthetic photon flux. Plant Growth Regul. 49, 137–146. doi: 10.1007/s10725-006-9003-z

[ref3] Augustin J. M. Drok S. Shinoda T. Sanmiya K. Nielsen J. K. Khakimov B.. (2012). UDP glycosyltransferases from the UGT73C subfamily in *Barbarea vulgaris* catalyze sapogenin 3-O-glucosylation in saponin-mediated insect resistance. Plant Physiol. 160, 1881–1895. doi: 10.1104/pp.112.202747, PMID: 23027665 PMC3510118

[ref4] Bennett R. N. Wallsgrove R. M. (1994). Secondary metabolites in plant defence mechanisms. New Phytol. 127, 617–633. doi: 10.1111/j.1469-8137.1994.tb02968.x 33874382

[ref5] Bowman S. M. Free S. J. (2006). The structure and synthesis of the fungal cell wall. Bioessays 28, 799–808. doi: 10.1002/bies.20441 16927300

[ref6] Chen O. Deng L. Ruan C. Yi L. Zeng K. (2021). *Pichia galeiformis* induces resistance in postharvest citrus by activating the phenylpropanoid biosynthesis pathway. J. Agric. Food Chem. 69, 2619–2631. doi: 10.1021/acs.jafc.0c06283, PMID: 33594880

[ref7] Cheng C. Zhao Y. Li H. He F. Cao S. Yang X.. (2017). Control effect of HSAF from *Lysobacter enzymogenes* OH11 on pear valsa canker. Chinese J. Biol. Control 33, 114–120. doi: 10.16409/j.cnki.2095-039x.2017.01.016

[ref8] Daud N. S. Rosli M. A. Azam Z. M. Othman N. Z. Sarmidi M. R. (2019). *Paenibacillus polymyxa* bioactive compounds for agricultural and biotechnological applications. Biocatal. Agric. Biotechnol. 18:101092. doi: 10.1016/j.bcab.2019.101092

[ref9] Du N. Shi L. Yuan Y. Sun J. Shu S. Guo S. (2017). Isolation of a potential biocontrol agent *Paenibacillus polymyxa* NSY50 from vinegar waste compost and its induction of host defense responses against *Fusarium* wilt of cucumber. Microbiol. Res. 202, 1–10. doi: 10.1016/j.micres.2017.04.013, PMID: 28647117

[ref10] Fan H. Zeng L. Yang P. Guo Z. Bai T. (2016). First report of banana soft rot caused by *Klebsiella variicola* in China. Plant Dis. 100:517. doi: 10.1094/PDIS-05-15-0586-PDN

[ref11] Geng D. Shen X. Xie Y. Yang Y. Bian R. Gao Y.. (2020). Regulation of phenylpropanoid biosynthesis by MdMYB88 and MdMYB124 contributes to pathogen and drought resistance in apple. Hortic. Res. 7:102. doi: 10.1038/s41438-020-0324-2, PMID: 32637130 PMC7327078

[ref12] Glickmann E. Dessaux Y. (1995). A critical examination of the specificity of the Salkowski reagent for indolic compounds produced by phytopathogenic bacteria. Appl. Environ. Microbiol. 61, 793–796. doi: 10.1128/aem.61.2.793-796.1995, PMID: 16534942 PMC1388360

[ref13] Holt J. G. Krieg N. R. Sneath P. H. Staley J. T. Williams S. T. (1994). Bergey’s manual of determinative bacteriology. 9th Edn. Baltimor: William & Wilkins.

[ref14] Hong C. E. Kwon S. Y. Park J. M. (2016). Biocontrol activity of *Paenibacillus polymyxa* AC-1 against *pseudomonas syringae* and its interaction with *Arabidopsis thaliana*. Microbiol. Res. 185, 13–21. doi: 10.1016/j.micres.2016.01.004, PMID: 26946374

[ref15] Hu Y. Gai Y. Yin L. Wang X. Feng C. Feng L.. (2010). Crystal structures of a *Populus tomentosa* 4-coumarate: CoA ligase shed light on its enzymatic mechanisms. Plant Cell 22, 3093–3104. doi: 10.1105/tpc.109.072652, PMID: 20841425 PMC2965553

[ref16] Jordá T. Puig S. (2020). Regulation of ergosterol biosynthesis in *Saccharomyces cerevisiae*. Genes 11:795. doi: 10.3390/genes11070795, PMID: 32679672 PMC7397035

[ref17] Kim Y. S. Balaraju K. Jeon Y. (2016). Biological control of apple anthracnose by *Paenibacillus polymyxa* APEC128, an antagonistic rhizobacterium. Plant Pathology J. 32, 251–259. doi: 10.5423/PPJ.OA.01.2016.0015, PMID: 27298600 PMC4892821

[ref18] Liu H. Li Y. Ge K. Du B. Liu K. Wang C.. (2021). Interactional mechanisms of *Paenibacillus polymyxa* SC2 and pepper (*Capsicum annuum* L.) suggested by transcriptomics. BMC Microbiol. 21, 70–16. doi: 10.1186/s12866-021-02132-2, PMID: 33663386 PMC7931354

[ref19] Liu R. Li J. Zhang F. Zheng D. Chang Y. Xu L.. (2021). Biocontrol activity of *Bacillus velezensis* D4 against apple Valsa canker. Biol. Control 163:104760. doi: 10.1016/j.biocontrol.2021.104760

[ref20] Livak K. Schmittgen T. (2001). Analysis of relative gene expression data using real-time quantitative PCR and the 2^−ΔΔCT^ method. Methods 25, 402–408. doi: 10.1006/meth.2001.1262 11846609

[ref21] Mei L. Liang Y. Zhang L. Wang Y. Guo Y. (2014). Induced systemic resistance and growth promotion in tomato by an indole-3-acetic acid-producing strain of *Paenibacillus polymyxa*. Ann. Appl. Biol. 165, 270–279. doi: 10.1111/aab.12135

[ref22] Mohd Din A. R. J. Rosli M. A. Mohamad Azam Z. Othman N. Z. Sarmidi M. R. (2020). *Paenibacillus polymyxa* role involved in phosphate solubilization and growth promotion of *Zea mays* under abiotic stress condition. Proc. Natl. Acad. Sci., India, Sect. B Biol. Sci. 90, 63–71. doi: 10.1007/s40011-019-01081-1

[ref23] Morrissey J. P. (2009). Biological activity of defence-related plant secondary metabolites. Plant-derived natural products. New York: Springer, 283–299.

[ref24] Nemesio-Gorriz M. Hammerbacher A. Ihrmark K. Källman T. Olson Å. Lascoux M.. (2016). Different alleles of a gene encoding leucoanthocyanidin reductase (*PaLAR3*) influence resistance against the fungus *Heterobasidion parviporum* in *Picea abies*. Plant Physiol. 171, 2671–2681. doi: 10.1104/pp.16.00685, PMID: 27317690 PMC4972290

[ref25] Park J. K. Kim J. D. Park Y. I. Kim S. K. (2012). Purification and characterization of a 1, 3-*β*-D-glucanase from *Streptomyces torulosus* PCPOK-0324. Carbohydr. Polym. 87, 1641–1648. doi: 10.1016/j.carbpol.2011.09.058

[ref26] Piasecka A. Jedrzejczak-Rey N. Bednarek P. (2015). Secondary metabolites in plant innate immunity: conserved function of divergent chemicals. New Phytol. 206, 948–964. doi: 10.1111/nph.13325, PMID: 25659829

[ref27] Puri A. Padda K. P. Chanway C. P. (2016). Evidence of nitrogen fixation and growth promotion in canola (*Brassica napus* L.) by an endophytic diazotroph *Paenibacillus polymyxa* P2b-2R. Biol. Fertil. Soils 52, 119–125. doi: 10.1007/s00374-015-1051-y

[ref28] Rahimi S. Kim J. Mijakovic I. Jung K. H. Choi G. Kim S. C.. (2019). Triterpenoid-biosynthetic UDP-glycosyltransferases from plants. Biotechnol. Adv. 37:107394. doi: 10.1016/j.biotechadv.2019.04.016, PMID: 31078628

[ref29] Samanta A. Das G. Das S. K. (2011). Roles of flavonoids in plants. Carbon 100, 12–35.

[ref30] Shadle G. L. Wesley S. V. Korth K. L. Chen F. Lamb C. Dixon R. A. (2003). Phenylpropanoid compounds and disease resistance in transgenic tobacco with altered expression of l-phenylalanine ammonia-lyase. Phytochemistry 64, 153–161. doi: 10.1016/S0031-9422(03)00151-1, PMID: 12946414

[ref31] Slama H. B. Cherif-Silini H. Chenari Bouket A. Qader M. Silini A. Yahiaoui B.. (2019). Screening for *Fusarium* antagonistic bacteria from contrasting niches designated the endophyte *Bacillus halotolerans* as plant warden against *Fusarium*. Front. Microbiol. 9:3236. doi: 10.3389/fmicb.2018.03236, PMID: 30687252 PMC6336696

[ref32] Song X. Han M. He F. Wang S. Li C. Wu G.. (2020). Antifungal mechanism of dipicolinic acid and its efficacy for the biocontrol of pear Valsa canker. Front. Microbiol. 11:958. doi: 10.3389/fmicb.2020.00958, PMID: 32508781 PMC7251846

[ref33] Thimmappa R. Geisler K. Louveau T. O’Maille P. Osbourn A. (2014). Triterpene biosynthesis in plants. Annu. Rev. Plant Biol. 65, 225–257. doi: 10.1146/annurev-arplant-050312-120229 24498976

[ref34] Ullah C. Unsicker S. B. Fellenberg C. Constabel C. P. Schmidt A. Gershenzon J.. (2017). Flavan-3-ols are an effective chemical defense against rust infection. Plant Physiol. 175, 1560–1578. doi: 10.1104/pp.17.00842, PMID: 29070515 PMC5717727

[ref35] Vanholme R. Cesarino I. Rataj K. Xiao Y. Sundin L. Goeminne G.. (2013). Caffeoyl shikimate esterase (CSE) is an enzyme in the lignin biosynthetic pathway in *Arabidopsis*. Science 341, 1103–1106. doi: 10.1126/science.1241602, PMID: 23950498

[ref36] Wang X. Zang R. Yin Z. Kang Z. Huang L. (2014). Delimiting cryptic pathogen species causing apple Valsa canker with multilocus data. Ecol. Evol. 4, 1369–1380. doi: 10.1002/ece3.1030, PMID: 24834333 PMC4020696

[ref37] Yadav V. Wang Z. Wei C. Amo A. Ahmed B. Yang X.. (2020). Phenylpropanoid pathway engineering: an emerging approach towards plant defense. Pathogens 9:312. doi: 10.3390/pathogens9040312, PMID: 32340374 PMC7238016

[ref38] Yang X. Zhang L. Xiang Y. Du L. Huang X. Liu Y. (2020). Comparative transcriptome analysis of *Sclerotinia sclerotiorum* revealed its response mechanisms to the biological control agent, *Bacillus amyloliquefaciens*. Sci. Rep. 10, 12576–12512. doi: 10.1038/s41598-020-69434-9, PMID: 32724140 PMC7387486

[ref39] Yin Z. Liu H. Li Z. Ke X. Dou D. Gao X.. (2015). Genome sequence of Valsa canker pathogens uncovers a potential adaptation of colonization of woody bark. New Phytol. 208, 1202–1216. doi: 10.1111/nph.13544, PMID: 26137988

[ref40] Yu F. Feng Y. Han J. Sheng Q. Sun L. Luo M. (2021). Screening of antagonistic bacteria against *Valsa pyri* from agricultural plant Jiaosu and their control effects on pear canker. J. Agric. Sci. Technol. 23, 125–135. doi: 10.13304/j.nykjdb.2021.0226

[ref41] Yu G. Wang L. Han Y. He Q. (2012). ClusterProfiler: an R package for comparing biological themes among gene clusters. OMICS 16, 284–287. doi: 10.1089/omi.2011.0118, PMID: 22455463 PMC3339379

[ref42] Yuan H. Shi B. Huang T. Zhou Z. Wang L. Hou H.. (2021). Biological control of pear Valsa canker caused by *Valsa pyri* using *Penicillium citrinum*. Horticulturae 7:198. doi: 10.3390/horticulturae7070198

[ref43] Yuan H. Shi B. Wang L. Huang T. Zhou Z. Hou H.. (2022). Isolation and characterization of *Bacillus velezensis* strain P2-1 for biocontrol of apple postharvest decay caused by *Botryosphaeria dothidea*. Front. Microbiol. 12:808938. doi: 10.3389/fmicb.2021.808938, PMID: 35058916 PMC8764377

[ref44] Zhai Y. Zhu J. Tan T. Xu J. Shen A. Yang X.. (2021). Isolation and characterization of antagonistic *Paenibacillus polymyxa* HX-140 and its biocontrol potential against *Fusarium* wilt of cucumber seedlings. BMC Microbiol. 21, 75–12. doi: 10.1186/s12866-021-02131-3, PMID: 33676418 PMC7936408

[ref45] Zhang F. Li X. Zhu S. Ojaghian M. R. Zhang J. (2018). Biocontrol potential of *Paenibacillus polymyxa* against *Verticillium dahliae* infecting cotton plants. Biol. Control 127, 70–77. doi: 10.1016/j.biocontrol.2018.08.021

[ref46] Zhang Q. Xing C. Li S. He L. Qu T. Chen X. (2021). In vitro antagonism and biocontrol effects of *Paenibacillus polymyxa* JY1-5 against *Botrytis cinerea* in tomato. Biol. Control 160:104689. doi: 10.1016/j.biocontrol.2021.104689

[ref47] Zhou C. Guo J. Zhu L. Xiao X. Xie Y. Zhu J.. (2016). *Paenibacillus polymyxa* BFKC01 enhances plant iron absorption via improved root systems and activated iron acquisition mechanisms. Plant Physiol. Biochem. 105, 162–173. doi: 10.1016/j.plaphy.2016.04.025, PMID: 27105423

