# Supplementary figures and images for: Biocontrol activity and action mechanism of Paenibacillus polymyxa strain Nl4 against pear Valsa canker caused by Valsa pyri
Source: Front Microbiol. 2022 Jul 22;13:950742. doi: 10.3389/fmicb.2022.950742 (PMC9354778; doi:10.3389/fmicb.2022.950742)

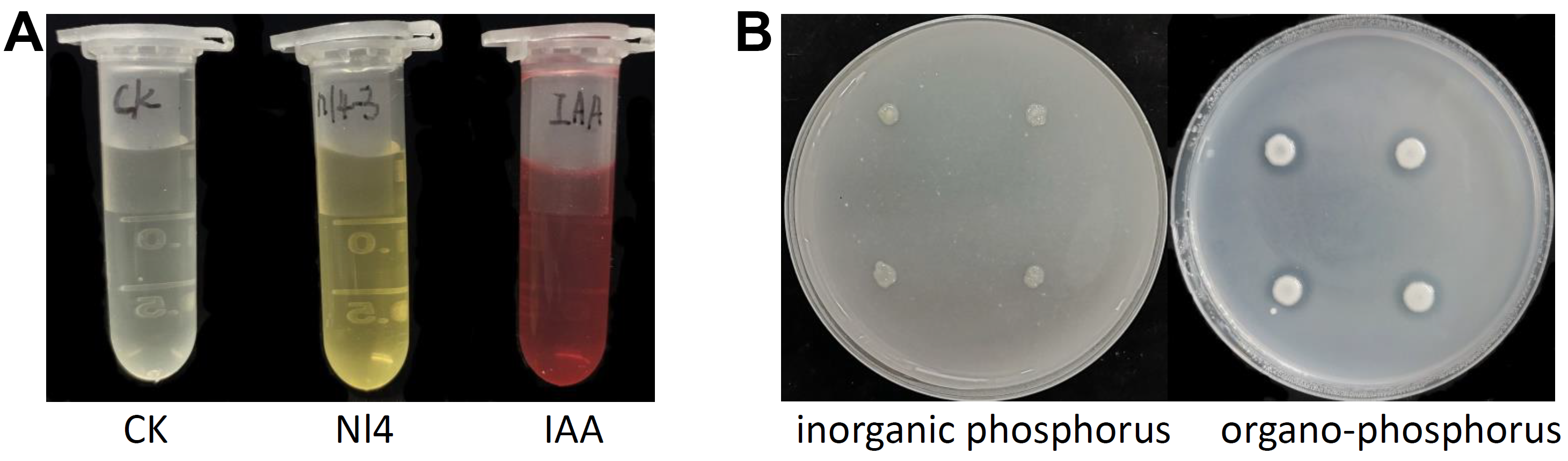

Supplement: Supplementary Figure 1 — The endophyte strain Nl7 did not show antagonistic activity against V. pyri. [file Data_Sheet_1.ZIP › Supplementary Figure 2.tif]

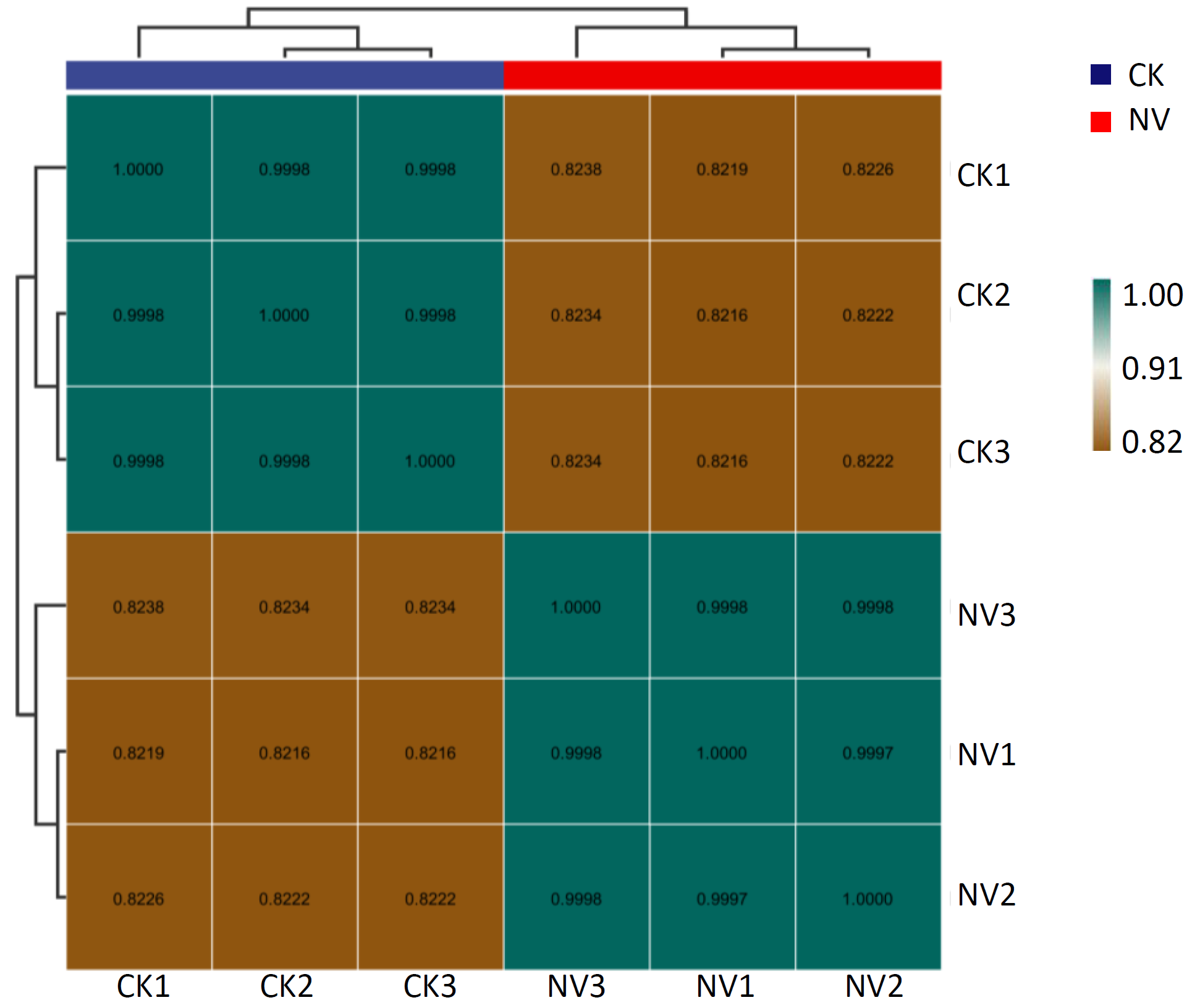

Supplement: Supplementary Figure 1 — The endophyte strain Nl7 did not show antagonistic activity against V. pyri. [file Data_Sheet_1.ZIP › Supplementary Figure 3.tif]

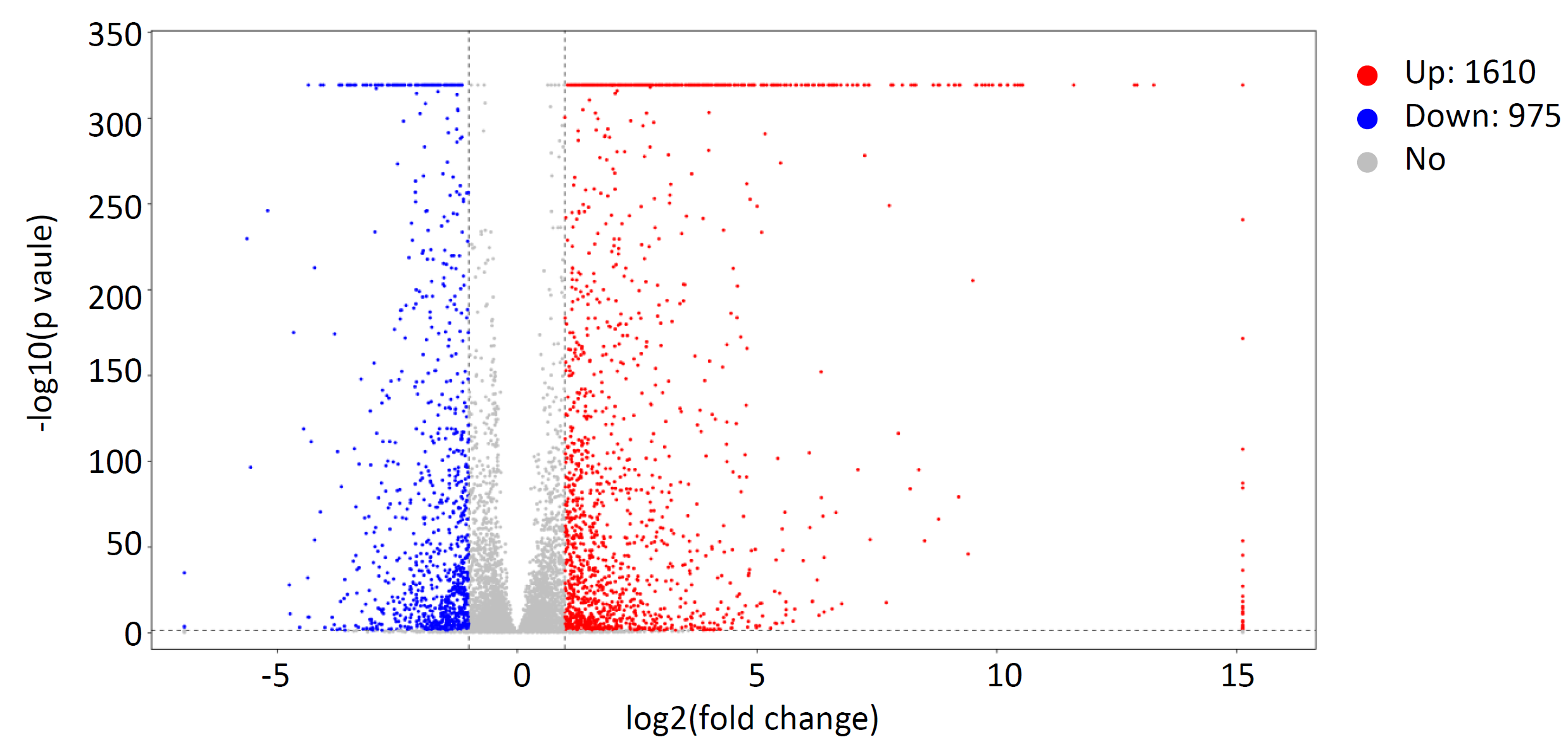

Supplement: Supplementary Figure 1 — The endophyte strain Nl7 did not show antagonistic activity against V. pyri. [file Data_Sheet_1.ZIP › Supplementary Figure 4.tif]

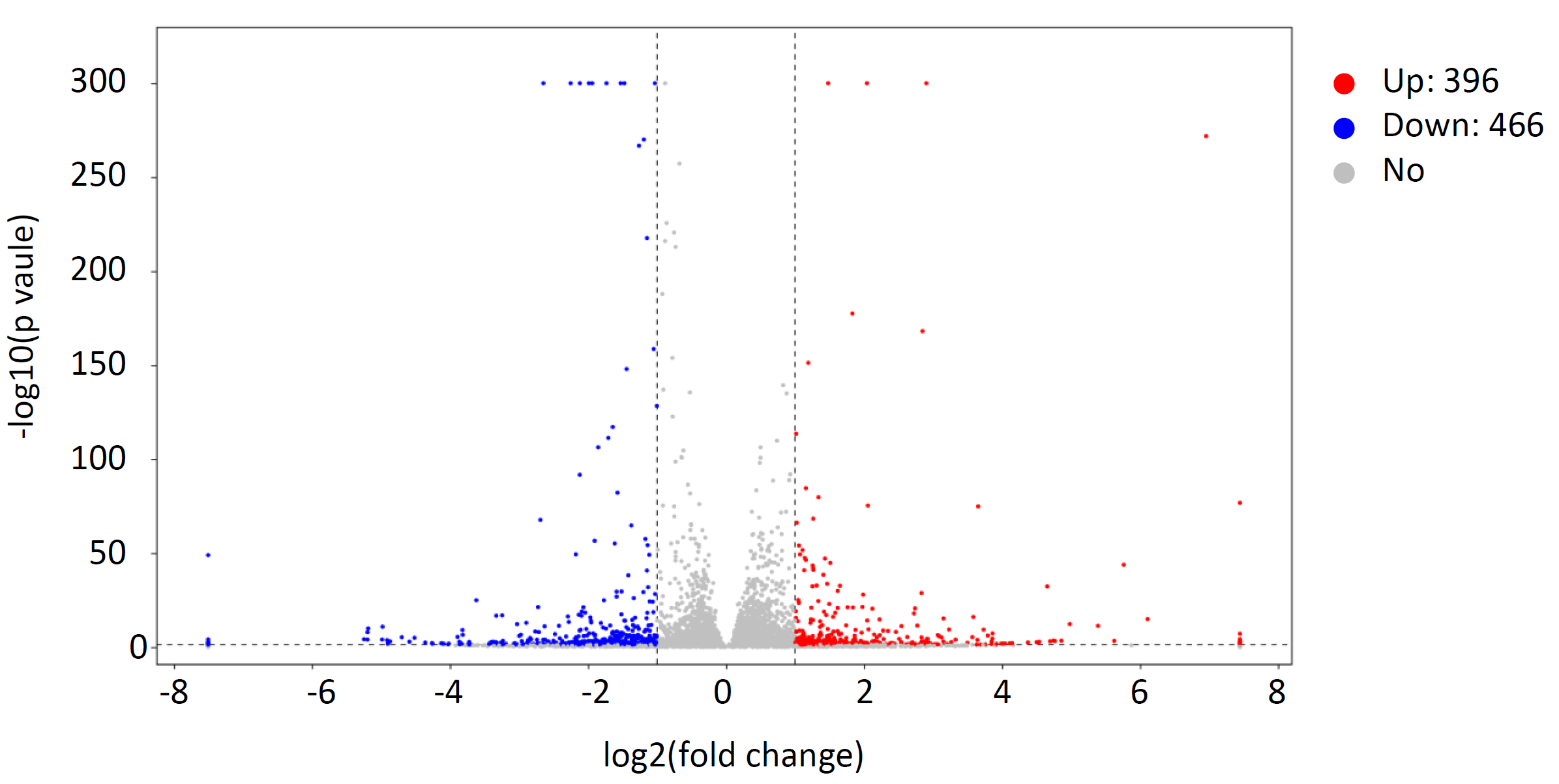

Supplement: Supplementary Figure 1 — The endophyte strain Nl7 did not show antagonistic activity against V. pyri. [file Data_Sheet_1.ZIP › Supplementary Figure 5.tif]

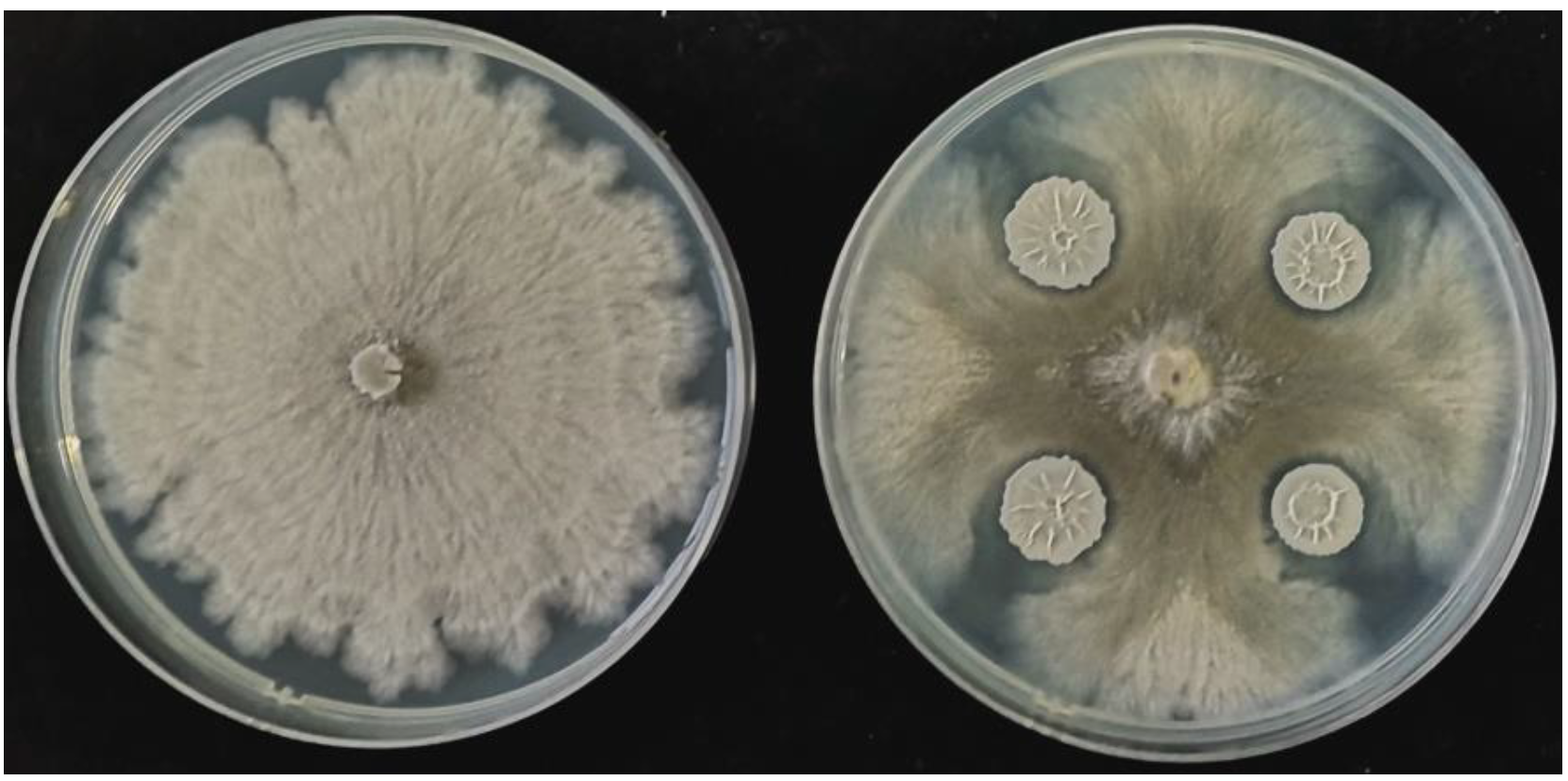

Supplement: Supplementary Figure 1 — The endophyte strain Nl7 did not show antagonistic activity against V. pyri. [file Data_Sheet_1.ZIP › Supplementary Figure 1.tif]
